# Effect of Polyol Plasticizers on the Physicochemical, Mechanical, and Microstructural Properties of Films from Chayote (*Sechium edule*) Peels

**DOI:** 10.1021/acsomega.5c07160

**Published:** 2026-02-04

**Authors:** Laura Arroyo-Esquivel, Víctor M. Jiménez, Fabián Vásquez-Sancho, Esteban Avendaño-Soto, Patricia Esquivel

**Affiliations:** † Escuela de Tecnología de Alimentos, 27915 Universidad de Costa Rica, San Pedro 11501-2060, Costa Rica; ‡ Centro Nacional de Ciencia y Tecnología de Alimentos (CITA), Universidad de Costa Rica, San Pedro 11501-2060, Costa Rica; § Centro para Investigaciones en Granos y Semillas (CIGRAS) and Instituto de Investigaciones Agrícolas (IIA), Universidad de Costa Rica, San Pedro 11501-2060, Costa Rica; ∥ Escuela de Física and Centro de Investigación en Ciencia e Ingeniería de Materiales, Universidad de Costa Rica, San Pedro 11501-2060, Costa Rica

## Abstract

This study aimed to develop biopolymer films (BFs) from immature chayote [*Sechium edule* (Jacq.) Sw.] Quelite variety fruit peels using glycerol, sorbitol, and ethylene glycol as plasticizers at three concentrations (0.05, 0.06, and 0.07 mol/L) using the casting method. The BFs were evaluated at two time points (day 1 and day 30 after casting) to assess their performance, stability, and potential food packaging applications. They were homogeneous, continuous, and exhibited colors ranging from cream to light brown, and had an average thickness of 198 μm. The average solubilities in water, acid, and base were 49.57%, 48.51%, and 65.15%, respectively, while the average moisture content across all treatments was 25.52%. The percentage of elongation increased from 4.8% to 6.8% with higher plasticizer concentrations. No significant effects of plasticizer type, concentration, or storage time were observed on tensile strength, with average values of 0.22 MPa. Water vapor permeability (WVP) ranged from 0.72 to 16.07 g·Pa^–1^·s^–1^·m^–2^·10^–7^, with significant differences among plasticizers. The films displayed a glass transition temperature of 63.29 °C and a melting temperature of 154.26 °C, indicating a homogeneous and stable matrix. These findings highlight the potential of chayote peel-based BFs as sustainable materials for food packaging applications.

## Graphical abstract

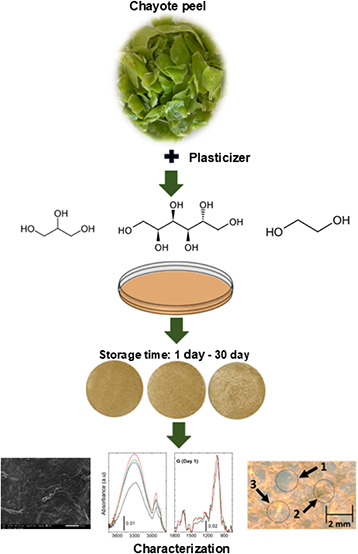

## 1 Introduction

The use of plastic packaging raises significant environmental and health concerns. These petroleum-based materials are slow to degrade, leading to long-term contamination of soil and oceans. Furthermore, they break down into microplastics that harm marine life and enter the food chain, posing risks to human health. Their production also contributes to climate change through the release of greenhouse gases.^1^ In this context, biopolymer films (BFs), especially those based on polysaccharides derived from agro-industrial byproducts, emerge as promising alternatives to mitigate the drawbacks of conventional plastics. Various studies have highlighted the growing interest in using polysaccharide-rich agricultural wastes as raw materials for the development of biobased packaging.^2^ One of the fundamental properties of polysaccharides is their natural film-forming ability, which can be further enhanced by incorporation of plasticizers, thus improving the properties of BFs for sustainable packaging applications.^3^ Additionally, the valorization of agro-industrial wastes, such as fruit and vegetable peels, supports the achievement of several Sustainable Development Goals (SDGs), particularly SDG 3 (Good health and well-being) by reducing microplastic-related health risks, SDG 12 (Responsible production and consumption) through waste reduction and resource efficiency, SDG 13 (Climate action) by reducing greenhouse gas emissions, and SDG 14 (Life below water) by reducing plastic pollution in marine ecosystems.^4^

Chayote [*Sechium edule* (Jacq.) Sw.] (Cucurbitaceae) is an herbaceous, monoecious, climbing plant. Its fruits, tender leaves, and tuberous roots are consumed as vegetables and are also used in the food industry to produce baby foods, juices, sauces, and pastes. Chayote fruits have a wide variety of shapes (globular, ovoid, subovoid, pyriform), sizes (4.3–26.5 cm long, 3–11 cm wide), spine type and densities, as well as colors.^5^ Chayote cultivation has become a significant economic activity for many farmers in Mexico, Costa Rica, Brazil, and the Dominican Republic.^6^ Among the different chayote varieties, the most widely consumed and exported in Costa Rica, especially to the European Union, is the Quelite variety. It is characterized by a smooth, spineless surface, the absence of longitudinal grooves, a pear-like shape, light green color, a size between 10 and 12 cm, and a weight of approximately 350 to 450 g per fruit.^7^

During the postharvest processing and industrial transformation of the chayote fruit, the peels constitute the main byproduct, generating large amounts of organic waste that pose a disposal challenge for producers. Chayote peels represent approximately one-sixth of the total weight of the fruit,^8^ resulting in significant amounts of biomass that could be valorized as a source of polysaccharides with physicochemical and mechanical properties suitable for use as raw material in the development of BFs for biodegradable packaging. Chayote peels differ from other byproducts due to their distinctive polysaccharide composition (particularly cellulose, hemicellulose, pectin, and starch).^6^ Functionally, this composition leads to films with lower tensile strength but greater flexibility and moisture retention, since the matrix is intrinsically hydrated and interacts differently with plasticizers. This chemical and functional profile justifies our work as a novel contribution, as it evaluates how a specific balance of polysaccharides, different from previously studied residues, affects film formation and performance for potential food packaging applications.

However, despite the numerous benefits offered by BFs, these materials have some limitations, including low water resistance; the potential for undesirable reactions such as premature degradation, discoloration, swelling, or brittleness; and limited mechanical strength and stability over time. To address these challenges, the formulation of the BFs and their final properties are often optimized by adding plasticizers. The appropriate selection and concentration of plasticizers can enhance the mechanical performance of BFs as well as their water and oxygen permeability.^9,10^

Several studies have investigated the use of plasticizers to improve the functional properties of BFs derived from agro-industrial byproducts. Glycerol, sorbitol, and ethylene glycol are among the most commonly used plasticizers in the production of BFs for packaging.^11^ For instance, glycerol has been applied to BFs made from citrus fruit peels,^12^ avocado peels,^13^ and a variety of other peels including potato, quince, and orange.^14^ Additionally, glycerol has been used in BFs developed from palm date byproducts^15^ and coffee processing residues.^16^ On the other hand, polyethylene glycol has been used as a plasticizer in BFs derived from grapefruit peels.^17^

The optimal proportion of each plasticizer depends on factors such as the extraction method, the base material, and the intended application of the final product.^10^ To date, no studies have reported the use of chayote byproducts for the development of BFs. The novelty of our work lies not only in the use of a new raw material but in addressing how the chemical composition of chayote peels influences polymer–plasticizer interactions and film properties. This distinctive matrix raises scientific questions about film formation and stability that cannot be fully answered with other agro-industrial residues. Therefore, the objective of this research is to develop BFs using chayote peel as raw material and to evaluate the effect of three plasticizers (glycerol, sorbitol, and ethylene glycol) at three concentrations (0.05, 0.06, and 0.07 mol/L) on the BFs’ physicochemical and mechanical properties. These concentrations were selected as representative low-to-moderate ranges commonly reported in biopolymer film studies, allowing us to identify the threshold at which improvements in flexibility and mechanical integrity can be achieved without compromising film stability. Establishing this balance is particularly relevant for food packaging, where materials must combine adequate strength with barrier properties to ensure product protection and shelf life extension.

## 2 Experimental Section

### 2.1 Plant Material

Chayote [*Sechium edule* (Jacq.) Sw.] fruits of the Quelite variety were used in this study. This variety is characterized by a spineless, shiny, and smooth epidermis. Fruits at an unripe stage (form most commonly consumed in meals in Costa Rica), weighing between 300 and 500 g and with about 10 to 12 cm in length, whose tender tissue can be easily cut with a knife, were manually peeled. The peels were collected in three independent batches on different days in March 2022 from agro-industrial facilities located in Paraíso, Cartago, Costa Rica. Immediately after collection, the peels were placed in Ziploc-type plastic bags (2 kg per batch) and stored at −6 °C to prevent microbial degradation and enzymatic activity prior to processing (April 2022).

### 2.2 Processing Methods

The peels were thawed and disinfected by immersion in a 2.5% (v/v) HCl (37% w/w, ACS grade, Merck, Germany) solution for 30 min, followed by thorough rinsing with water. They were then cut into squares (0.5 × 0.5 cm), weighed (*P*1), mixed with water at a ratio of 2.5:1 (m/m), and boiled at 60 °C for 15 min with constant stirring (600–700 rpm). The resulting mixture was blended (KitchenAid Diamond, KSB1570SL, Ohio, USA) until completely homogeneous and then sieved through a fine-mesh strainer (mesh size #6). The filtrate was weighed (*P*2) and 0.0005% (m/m) natamycin was added to prevent fungal contamination. The raw material yield (%*Y*) was determined in triplicate following eq 1.

1 %Y=(P2/P1)×100

### 2.3 Characterization of Raw Material

The raw material used to produce the BFs was characterized as follows: (i) Determination of moisture and dry matter content at 60 °C,^18^ (ii) Acid Detergent Fiber (ADF) according to AOAC method 973.18, (iii) Neutral Detergent Fiber (NDF) according to AOAC method 2002.04, (iv) Lignin content within the ADF fraction according to AOAC method 973.18, and (v) Pectin content according to the methods published elsewhere.^19,20^

### 2.4 Experimental Design

A randomized block design was employed, structured around a 4 × 3 × 2 factorial arrangement to evaluate the effects of three experimental factors: type of plasticizer (no plasticizer/control, glycerol, sorbitol, and ethylene glycol), concentration of plasticizer [0.05, 0.06, and 0.07 mol/L (were selected according to references^21^)], and storage time (1 and 30 days). This configuration yielded a total of 20 distinct treatments. Each treatment was replicated across three independent chayote peel batches, which served as experimental blocks. These plasticizers were selected because they are commonly used in BFs production due to their proven compatibility with polysaccharide matrices and their regulatory approval for food-contact materials.^10^

On day 1, the parametric response variables measured included thickness, water solubility, acid solubility, base solubility, humidity, and water vapor permeability (WVP). Parameters assessed both on day 1 and after 30 days of storage included color (L*, a*, b*), % opacity, percentage of elongation at break (%*E*), tensile strength (TS), number of air bubbles, number of fibers and pigmented fibers, as well as glass transition temperature (Tg), melting temperature (Tm), and enthalpy of fusion (ΔHm). Descriptive analyses were also conducted using differential scanning calorimetry (DSC), X-ray diffraction (XRD), Fourier-transform infrared spectroscopy with attenuated total reflection (FTIR-ATR), and scanning electron microscopy (SEM).

### 2.5 BFs Casting

BFs were prepared by the casting method, following the general procedures described by Escobar-Guadarrama^21^ and Donkor et al.^22^ A total of ten film formulations were prepared: one control (without plasticizer) and nine treatments combining the three types of plasticizers at three concentration levels, as described in Section 2.4. For each formulation, 17 g of the previously processed raw material (see Section 2.2) was poured into sterile 90 mm polystyrene Petri dishes. The BFs were then dried in a forced-air oven at 55 °C, for 12 h.

### 2.6 Characterization of BFs

#### 2.6.1 Visual and Tactile Analysis

The BFs were visually and manually examined to assess their continuity (defined as the absence of cracks or breaks after drying) and their flexibility, understood as the ability to be handled without tearing. This qualitative evaluation was based on the methodology described by Mali et al.^23^

#### 2.6.2 Color

Color measurements were made on each one of the BFs by averaging three readings per sample: one from the center and two from opposite edges. A ColorFlex EZ spectrophotometer (HunterLab, USA) was used, configured with a 10° viewing angle and D65 illuminant. The CIE-Lab color space was applied, where L* indicates lightness (ranging from 0 for black to 100 for white), a* reflects the red-green spectrum (negative values toward green, positive toward red), and b* represents the blue-yellow spectrum (negative toward blue, positive toward yellow). Based on these parameters, the hue angle (*h*°) and chroma (C*) were calculated using eqs 2 and 3, respectively.^24,25^

2 $$h^{{}^{\circ}}=\tan^{-1}\left(\frac{b^{*}}{a^{*}}\right)$$

3 $$C^{*}={\left(a^{*2}+b^{*2}\right)}^{\frac{1}{2}}$$

All measurements were performed in triplicate. To evaluate color stability over time, the total color difference (ΔE*) between day 1 and day 30 was calculated using eq 4:

4 $$\Delta E^{*}={[{\left(\Delta L^{*}\right)}^{2}+{\left(\Delta a^{*}\right)}^{2}+{\left(\Delta b^{*}\right)}^{2}]}^{\frac{1}{2}}$$

#### 2.6.3 Opacity

The opacity (*Op*) of the BFs was evaluated using a ColorFlex EZ spectrophotometer (HunterLab, USA) after calibration with standard white and black tiles to obtain reflectance values Yw and Yb, respectively. Each film sample was measured in triplicate under standardized conditions using a D65 illuminant and a 10° viewing angle. The percentage Op was then calculated according to eq 5:^25,26^

5 Op=Yw/Yb×100

#### 2.6.4 Quantity of Air Bubbles, Fibers, and Pigmented Fibers

The number of air bubbles, fibers, and pigmented fibers present in the films was quantified using ImageJ Fiji software.^27^ High-resolution scans were obtained using an Epson V700 scanner (Nagano, Japan) set to 1600 dpi. Analyses were conducted in triplicate on defined 1 cm^2^ areas of each one of the BFs to ensure consistent evaluation of microstructural features.

#### 2.6.5 Thickness

The thickness of each film was determined with a Mitutoyo digital thickness gauge, model JIS B 7502 (Nagoya, Japan). Five measurements were taken at randomly selected points across the film surface, and their average was reported as the final thickness value.^28^

#### 2.6.6 Solubility

Water solubility was assessed in triplicate using circular BFs samples (1 cm diameter) according to the procedure described by Gontard and Guilbert.^29^ The initial mass (mi) of each sample was recorded prior to immersion in 25 mL of distilled water with constant stirring (150 rpm) at 25 °C for 1 h using a magnetic stirrer. After incubation, the undissolved film residues were collected, dried in a forced-air circulation oven at 105 °C for 24 h, allowed to cool, and then weighed to obtain the final mass (mf). The film solubility (%*S*) was calculated using eq 6.^24^

6 %S=(mi−mf)/mi×100

Solubility under acidic and basic conditions was determined by the same procedure, substituting distilled water with 1 N HCl and 1 N NaOH (NaOH, pellets, ≥98% purity, Sigma-Aldrich, USA) solutions, respectively.

#### 2.6.7 Moisture

The moisture content of the BFs was determined using the oven-drying method described in AOAC 950.46.^19^ Samples were dried at 105 °C until reaching a constant weight, and the moisture content was calculated based on the weight loss during drying.

#### 2.6.8 Mechanical Properties

TS and %*E* were determined in triplicate using a dynamic mechanical analyzer (RSA G2, TA Instruments, New Castle, USA) based on a modified version of ASTM D 882–18 standard method.^30^ BFs were cut into strips 2.5 cm in length and 0.5 cm in width, and mounted between grips with an initial spacing of 2 cm. The test was performed at 25 °C with a crosshead speed of 1 mm s^–1^ for a maximum of 60 s. TS, expressed in MPa, was calculated using eq 7, where Fm is the maximum force at break (*N*) and *A* is the cross-sectional area of the BFs (m^2^). %*E* was determined using eq 8, where do is the initial grip separation (cm) and dr is the distance between the grips at the point of rupture (cm).

7 TS=Fm/A

8 %E=(dr−do)/do×100

#### 2.6.9 WVP

WVP was evaluated in triplicate using the desiccant method according to ASTM E96/E96M-10,^31^ with methodological adaptations based on Rincón et al.^32^

#### 2.6.10 DSC

Tg, Tm, and ΔHm of the BFs were analyzed using a Discovery DSC 250 (TA Instruments, New Castle, USA). Samples weighing between 6 and 10 mg were placed in hermetically sealed aluminum pans and conditioned at 25 °C under 50% relative humidity prior to analysis. Measurements were performed under an ultrahigh purity (UHP) nitrogen atmosphere with both purge and balance flow rates set at 50 mL min^–1^. The temperature program started at 10 °C and increased at a rate of 10 °C min^–1^ until 250 °C was reached. Atmospheric air was used as the reference, following the procedure described by de Andrade et al.^33^

#### 2.6.11 XRD

XRD analysis was performed using an Empyrean X-ray diffractometer (Malvern Panalytical, The Netherlands). The instrument was configured with a copper anode operated at 45 kV and 40 mA, using Bragg–Brentano geometry and a GaliPIX3D detector. Data were collected with a step size of 0.01 and an integration time of 1 s per step. Samples were mounted on a reflection-transmission spinner rotating at one revolution per second.

#### 2.6.12 FTIR-ATR

The FTIR-ATR spectra of the BFs were recorded using a Frontier spectrometer (PerkinElmer, Waltham, MA, USA), scanning in the range of 4000 to 550 cm^–1^. The analysis was carried out following the general procedure described by Oliveira da Silva et al.,^26^ with adjustments as needed for sample handling and baseline correction.

#### 2.6.13 SEM

The surface morphology of the BFs was analyzed using a JSM-IT500-LA SEM equipped with an energy-dispersive X-ray (EDX) analyzer (JEOL, Tokyo, Japan). Observations were made at an acceleration voltage of 5 kV and a working distance of 10 mm. Aluminum cylinders (12 × 10 mm, lathe-finished) were used as holders to mount the BFs prior to SEM analysis. Elemental composition was determined through EDX, which was conducted in high vacuum mode with an incident beam energy of 20.0 kV, a working distance of 10–14 mm, and a dead time maintained between 5% and 10%.^33^

#### 2.6.14 Statistical Analysis

All statistical procedures were conducted using JMP Pro 15 software (SAS Institute Inc., Cary, NC, USA). Analysis of variance was applied to evaluate differences among treatments, followed by Tukey’s posthoc test to identify statistically significant differences at a 95% confidence level (*p* < 0.05). Results for BFs characterization are presented as mean ± standard deviation, based on triplicate determinations.

## 3 Results and Discussion

### 3.1 Determination of Yield and Characterization of Raw Material

Approximately 546 ± 10 g of blended and sieved raw material was obtained from 1 kg of chayote peels, corresponding to a yield of 55 ± 2%. This value is comparable to the yields reported for other fruit byproducts, such as 59% from bocaiuva (*Acrocomia aculeata* Jacq.) flour^26^ and 62% from pitahaya (*Hylocereus* sp.) peels.^24^

Analysis of the raw material showed a high moisture content of 93.2 g/100 g (Table 1), which is consistent with the findings of Bellur and Prakash,^34^ who reported a moisture content of 94 g/100 g in chayote peels. Unlike studies using powdered peels such as citrus,^12^ avocado,^13^ fresh white grapefruit,^17^ quince, potato, and orange,^14^ the starting material in this study was a gel. This gel-like consistency probably facilitated the film formation in Petri dishes using the casting method.

**Table 1:** Chemical Characterization of Chayote Fruit Peels Used to Produce Biopolymer Films (Control)^a^

Proximal analyses	Average contents (g/100 g)
Humidity at 60 °C (DM^b^)	93.2 ± 0.6 FM (6.75 ± 0.05)
Acid Detergent Fiber (ADF) (DM)	26.7 ± 1.9
Neutral Detergent Fiber (NDF) (DM)	39.3 ± 1.9
Lignin Acid Detergent (DM)	3.9 ± 0.8
Pectins	6.0 ± 0.1
Starch (FM^c^)	0.368 ± 0.100

a Values are represented as average ± standard deviation (*n* = 3).

b DM (Dry matter).

c FM (Fresh matter).

The ADF fraction, which represents the fibrous portion containing cellulose, lignin, and heat-damaged proteins, showed a value of 26.7 g/100 g (Table 1). The NDF, which includes the ADF components plus hemicellulose, reached 39.3 g/100 g, indicating that approximately 12.6 g/100 g is hemicellulose. Other components such as pectin and small amounts of starch were also identified in the matrix. Overall, this composition suggests that the chayote peel-based material offers a rich mixture of structural polysaccharides and other constituents that may be beneficial for film formation. The interaction of these components may contribute to a more cohesive and stable network, improving the structural integrity and handling of the film during production. Similar effects have been observed in other plant-based systems, where such interactions have been associated with improved film properties.^35^

### 3.2 Physical Appearance

Demolding the control BFs was more difficult, as they adhered more strongly to the plates and were more brittle, compared to those containing polyols. This behavior supports the advantage of using plasticizers, which modify interactions within the polymer matrix, facilitating slippage and thereby improving flexibility and malleability.^36,37^ All BFs remained stable across treatments after 30 days of storage, with no visible cracks observed.

Homogeneous, continuous, and intact BFs were obtained using the employed method and after the drying process, displaying shades ranging from cream to light brown (Figure 1a). These natural tones could enhance consumer acceptance, particularly if intended for food packaging, as “earthy” colors like cream, brown, and green are often associated with sustainability.^38^ A slight whitening of the BFs toward a more uniform cream tone was observed during storage. Similar results have been reported for homogeneous and stable alginate-based films, such as those incorporating date palm pit (*Phoenix dactylifera* L., Variety Deglet Nour) byproducts^15^ and BFs made from powdered peels of four different citrus fruits [orange (*Citrus sinensis* L.), lime (*C. limon* L.), grapefruit (*C. paradisi* Macfad), and mandarin (*C. reticulata* Blanco)] combined with sodium alginate.^12^

**Figure 1:**
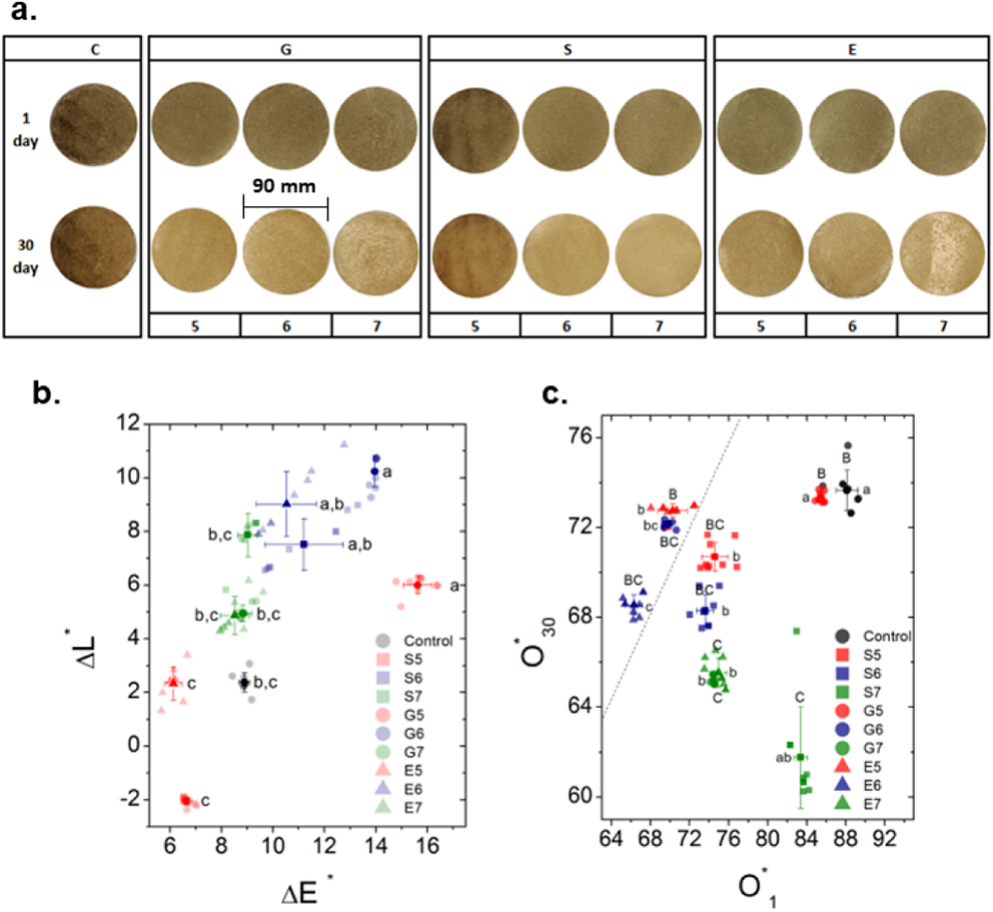
a. Biopolymer films produced with chayote peels after (1 and 30 days) using different plasticizers [glycerol (G), ethylene glycol (E), and sorbitol (S)] at three concentrations [0.05 (5), 0.06 (6), and 0.07 (7) mol/L] with a control (C). b. Total color difference (Δ*E*), [variation in the color parameters (L*, a*, b*)] and difference in luminosity (ΔL*) between days 1 and 30. Different letters (a-b) per parameters indicate significant differences with α = 0.05. Values are represented as average ± standard deviation (*n* = 3). c. Opacity [O* (%)] (1 and 30 days). Different lowercase letters (a-b) for day 1 indicate significant differences with α = 0.05. Different uppercase letters (A-B) for day 30 indicate significant differences with α = 0.05.

### 3.3 Color Parameters

Significant individual effects of the plasticizer type, concentration, and storage time, as well as their interactions (*p* < 0.0001), were observed on the color parameters (Table 2 and Figure 1b). The L* parameter revealed that control BFs were less luminous compared to those treated with plasticizers. The addition of plasticizers likely diluted the color, thereby raising the L* values,^39^ suggesting improved optical properties in plasticized films.^40^

**Table 2:** Color Parameters (CIE L*, a*, b*) of Biopolymer Films Produced with Chayote Peels Using Different Plasticizer Concentrations after 1 and 30 Days of Storage at Room Temperature^a^

Plasticizer	Concentration (mol/L)	Luminosity (L*)	Green-red chromaticity (a*)	Yellow-blue chromaticity (b*)	Chroma (C*)	Hue angle (*h*°)
Control	0	**39.2 ± 0.2f**	4.7 ± 0.1b	17 ± 0.4 g	17.6 ± 0.4f	**14.5 ± 0.4a**
Glycerol	0.05	46.5 ± 7.7d	**5.8 ± 0.1a**	**22.4 ± 0.1ef**	23.2 ± 0.1ab	13.2 ± 0.1b
Glycerol	0.06	53.2 ± 0.6a	3.5 ± 0.2d	25 ± 0.6ab	25.3 ± 0.6ab	7.3 ± 0.6e
Glycerol	0.07	49.2 ± 0.2c	3.9 ± 0.1c	23.8 ± 0.1cd	24.2 ± 0.1bcd	8.7 ± 0.1d
Sorbitol	0.05	47.8 ± 2.5d	**4.3 ± 0.4b**	**21.5 ± 2.2f**	22.1 ± 2.1e	11.03 ± 2.1c
Sorbitol	0.06	52.6 ± 0.8ab	3.3 ± 0.4de	25.9 ± 0.4a	26.2 ± 0.4a	6.8 ± 0.4e
Sorbitol	0.07	51.6 ± 0.5b	3.0 ± 0.6e	23.9 ± 0.6cd	24.1 ± 0.6cd	7.0 ± 0.6e
Ethylene glycol	0.05	44.7 ± 0.2e	**4.5 ± 0.2b**	**23.3 ± 0.2de**	23.8 ± 0.2cd	10.6 ± 0.2c
Ethylene glycol	0.06	51.5 ± 1.0b	3.3 ± 0.1de	25 ± 2.3ab	25.3 ± 0.1ab	7.2 ± 0.1e
Ethylene glycol	0.07	53.2 ± 0.5a	3.3 ± 0.3de	24.6 ± 1.8bc	24.9 ± 0.3bc	7.0 ± 0.3e

a Different lower-case letters per column indicate significant differences with α = 0.05. Values are represented as average ± standard deviation (*n* = 3).

After 30 days of storage, an increase in ΔL* was observed in both the control and most plasticized BFs, except for those formulated with sorbitol at 0.05 mol/L (Figure 1b). This change is likely due to structural modifications in the polymer matrix over time, which affect light reflection and, in turn, influence luminosity.^41^ Other contributing factors may include degradation of natural pigments and/or crystallization of polymer chains, both of which affect the luminosity of the BFs.^42^ An increase in the ΔL* can be considered favorable in food packaging applications as it indicates a more homogeneous and well-processed material with fewer impurities or structural irregularities.^41,43^

In addition, BFs with a positive green-red (a*) value were observed in the samples containing glycerol at 0.05 mol/L, followed by the control, sorbitol, and ethylene glycol at the same concentration (Table 2), indicating a slight shift toward red tones. In contrast, BFs with higher plasticizer concentrations (0.06 and 0.07 mol/L) showed lower a* values. These color differences could be attributed to the degradation of phenolic compounds and carotenoids naturally found in chayote peels.^6,56^

BFs containing plasticizers showed higher positive yellow-blue (b*) values compared to the control. Similar results were reported in films prepared from chia seed mucilage with glycerol, where b* values increased with the use of plasticizers.^44^ In addition, b* values at a concentration of 0.05 mol/L, regardless of the plasticizer used, were lower than those observed at 0.06 and 0.07 mol/L. This suggests that higher levels of plasticizer may enhance light reflection from the film surface.^45^

The chroma (C*) values, which represent color intensity, were higher in the BFs containing plasticizers. Among the sorbitol treatments, the highest C* value was observed at 0.06 mol/L, although it was not significantly different from glycerol at 0.05 and 0.06 mol/L or ethylene glycol at 0.06 mol/L. This can be considered positive because higher visual intensity enhances the appeal of the packaging by conveying freshness and quality, making it more attractive to consumers.^46^

Regarding hue (*h*°), the control BFs exhibited higher values, indicating a more yellowish tone. In contrast, the BFs containing plasticizers showed lower *h*° values, corresponding to more reddish tones. This shift toward yellow in the control BFs could be attributed to the degradation of natural pigments, such as flavonoids, chlorophylls, and carotenoids, present in the peels, as well as to interactions between the plasticizers and the film matrix, which affect the distribution of the chromophores responsible for the color.^47^

The total color difference (Δ*E*), which reflects the combined variation of color parameters (L*, a*, b*) between days 1 and 30, exceeded 2 for all treatments, indicating perceptible changes to the human eye.^48^ These changes during storage may be attributed to exposure to oxygen, light, and fluctuations in humidity.^49^ The most pronounced color changes were observed in BFs containing glycerol at 0.05 and 0.06 mol/L (Figure 1b). In BFs with glycerol, a highly hygroscopic plasticizer, humidity fluctuations may contribute to greater color instability.^50^

### 3.4 Opacity

The opacity of a material is a key factor in food packaging and preservation applications because exposure to light can adversely affect product quality. In this study, a significant effect of the type of plasticizer, its concentration, storage time, and their interactions (*p* < 0.0001) was observed on the opacity percentage (Figure 1c). The control BFs and those with glycerol at 0.05 mol/L showed the highest opacity values (around 80%). Hydrophilic plasticizers, such as the polyols tested in this work, are often reported to increase film opacity due to their interaction with polymer chains, which alters the film structure and the way it scatters light.^51^ However, in this case, the addition of plasticizers, except for glycerol at 0.05 mol/L, resulted in significantly lower opacity values compared to the control. This behavior can be explained by the ability of plasticizers to reduce crystallinity of the polymer matrix, by interrupting the crystallization process, resulting in increased amorphous regions within the material. This structural modification creates a more uniform optical environment that decreases light scattering and increases transparency.^52^ Notably, ethylene glycol at 0.06 mol/L produced the most pronounced reduction in opacity, with a 13% decrease compared to the control film.

The average opacity of the films decreased from 78.1 ± 2.8% on day 1 to 70.0 ± 1.3% after 30 days of storage. This reduction can be attributed to the degradation, molecular reorganization of film components, or structural changes in plasticizers and polymer chains that can occur over time. These modifications can alter the optical properties of the films by affecting light scattering mechanisms. Additionally, water evaporation during storage can modify the structure of the biofilms, potentially contributing to the observed changes in opacity. Additionally, water evaporation during storage can alter the structure of the BFs and promote increased crystallinity, further contributing to the observed reduction in opacity.^28^

The average opacity of the films was 74.0 ± 4.3%, which is considerably higher than that reported for other BFs, such as those made from *Cydonia oblonga* Miller byproducts (17–18%),^53^ chitosan combined with coconut byproducts (5.3–11.6%),^28^ and BFs based on pitahaya (*Hylocereus undatus* Britton & Rose) mucilage (10–11%).^54^ This high opacity is advantageous as it effectively protects the contents, such as food or other sensitive products, from exposure to light, particularly ultraviolet light, which can induce degradation, oxidation, color changes or loss of quality in the products.^51^ It also provides aesthetic appeal for organic or artisanal products targeting eco-conscious consumers. However, reduced transparency may discourage potential consumers who prefer to visually evaluate a product prior to purchase.^55^

### 3.5 Air Bubbles, Fibers, and Pigmented Fibers

No significant effect of plasticizer type, concentration, or storage time was observed on the number of bubbles, fibers, or pigmented areas in the BFs. In general, the number of bubbles and fibers provides information on the uniformity of the BFs; a higher number of these features correlates with lower uniformity.^26^ The BFs produced from chayote peels exhibited on average per cm^2^ 133.3 ± 12.7 bubbles, 13 ± 3 pigmented areas, and 94.5 ± 15.75 fiber units (Figure 2). These values show similarities with those measured in pitahaya (*Hylocereus* sp.) raw material for BFs production as reported by Arroyo-Esquivel et al.^24^ The presence of pigmented areas and fibers appeared to be intrinsic to the raw material, suggesting that these characteristics were not influenced by the plasticizers.

**Figure 2:**
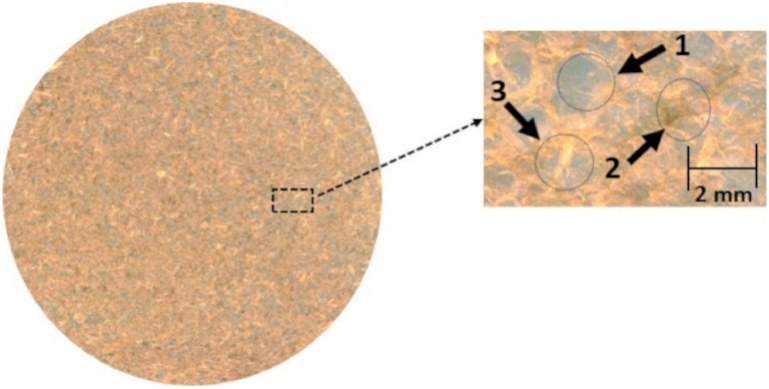
Scanned biopolymer films produced with chayote peel (zoom 75%). 1: bubbles, 2: fibers, and 3: pigmented fibers.

### 3.6 Characteristics of the BFs

#### 3.6.1 Thickness

The average thickness of the BFs was 198 ± 40 μm. No significant effect was observed from the type of plasticizer (*p* = 0.8189), its concentration (*p* = 0.8371), or the interaction between both factors (*p* = 0.9996). This result suggests that the plasticizer levels applied did not significantly alter the film volume. Consistent with the present findings, Bykov et al.^56^ reported no significant effect of plasticizer concentration on the thickness of films made from apple raw material (0.28–0.33 mm). This lack of effect may be attributed to the relatively low plasticizer concentrations used, which did not significantly impact the volume of the BFs. In contrast, Sanyang et al.^57^ observed a significant increase in film thickness with increasing plasticizer concentrations (15%, 30%, and 45%), regardless of the type used (glycerol or sorbitol); however, these concentrations were notably higher than those applied in the present study.

#### 3.6.2 Solubility

The average values obtained for solubility in water, acid, and base were 49.57 ± 9.92%, 48.51 ± 12.62%, and 65.15 ± 10.54%, respectively. No significant effect (*p* ≥ 0.05) of the type of plasticizer, its concentration, or the interaction between the two factors was observed on any of these variables. However, other studies have reported significant effects of both plasticizer type and concentration. Unlike the present study, those works used higher concentrations and broader ranges of plasticizers -for example, mung bean starch films with glycerol (0–30%) and sorbitol (0–60%),^58^ and blue corn films with glycerol and sorbitol (0–40%).^59^ In these cases, increases in water solubility were associated with higher plasticizer concentration, which was attributed to the ability of plasticizers to disrupt interactions between biopolymer chains, favoring plasticizer–polymer interactions. These interactions reduce the cohesion of the polymer matrix by decreasing the density of binding zones through hydrogen bonding.^58,60^

Interestingly, even at concentrations similar to those used in the present study, the same plasticizers had a significant effect on the solubility of pitahaya BFs.^24^ This contrast suggests that the lack of significant changes in the solubility of chayote BFs may be due to matrix-plasticizer interactions that are less pronounced or less favorable in chayote-based systems than in pitahaya-based matrices. Such differences may result from the intrinsic properties of the respective raw materials, including molecular composition and interaction potential.

The solubility of BFs is a key parameter as it directly influences their functionality, compatibility for different types of food, migration of film components into the food, and biodegradability, among others.^61^ Chayote-based BFs exhibited similar solubility in water and acid, both of which were lower than the solubility observed in alkaline media. In comparison, higher water solubilities were reported for films made from native Brazilian fruits (76.42–91.30%),^62^ and from poly­(vinyl alcohol)/gelatin blends (69.11–96.25%).^63^ In contrast, lower water solubility has been documented in sweet potato (*Ipomoea batatas* L.) starch films (7.51–18.15%),^60^ and in starch/TiO_2_ films (16.88–18.84%).^64^ In order for BFs and other structured materials to dissolve, cohesive forces within the polymer matrix must be overcome. Film solubility can be influenced by multiple structural and physicochemical factors including the composition and intrinsic properties of the raw materials used.^65^

#### 3.6.3 Moisture

Moisture content is a fundamental physical property of the films as it directly affects the quality of the final product through water loss or gain. In the chayote BFs, an average moisture value of 25.52 ± 7.27% was measured. Similar values (22.27 ± 3.28%) were reported by Arroyo-Esquivel et al.^24^ in BFs made from pitahaya peels and the same plasticizers and concentrations, and by Kurek et al.,^66^ who found moisture contents ranging from 15.0 to 25.0% in chitosan and pectin films. In contrast, Codina et al.^53^ reported higher moisture percentages (39.52–41.01%) in films made from quince byproducts.

In this study, no significant effect of the type of plasticizer, its concentration or their interaction was observed (*p* ≥ 0.05). Conversely, Dong et al.^11^ found significant effects of both variables on the moisture content of soybean polysaccharide films, with values ranging from 12.2% to 29.9%, likely due to the use of higher plasticizer concentrations (15%, 30%, and 45%). The absence of such effects in our study can be attributed to the use of lower concentrations, which were increased in 0.01 mol/L intervals. The absence of major changes in variables such as thickness, solubility, and moisture indicates that the matrix is relatively insensitive to small variations in plasticizer, which is a relevant finding for understanding the efficiency limits of plasticizers in this system.

#### 3.6.4 Mechanical Properties

Regarding the mechanical properties, no significant effect of plasticizer type or storage time (*p* ≥ 0.05) was observed on the %*E*. However, plasticizer concentration had a significant effect on this variable (*p* = 0.0081), with %*E* showing higher values at 0.07 mol/L than in the control BFs (Figure 3a). A similar trend was reported by Razavi et al.^67^ in sage seed gum films, where plasticizer concentration significantly influenced %*E*, whereas the type of plasticizer (glycerol or sorbitol) did not. This effect has been attributed to the plasticizer disrupting intermolecular interactions and increasing the free volume within the polymer matrix. As plasticizer concentration increases, more molecules are incorporated into the matrix, forming bonds that disrupt the polymer structure. This results in a more flexible and disordered material, thereby increasing %*E*.^60,67^ The lack of a significant effect of storage time (*p* < 0.5793) suggests that the elongation of the BFs is maintained for at least 30 days, likely due to effective plasticizer integration into the polymer matrix and resistance to time-dependent degradation mechanisms (e.g., phase separation, leaching).

**Figure 3:**
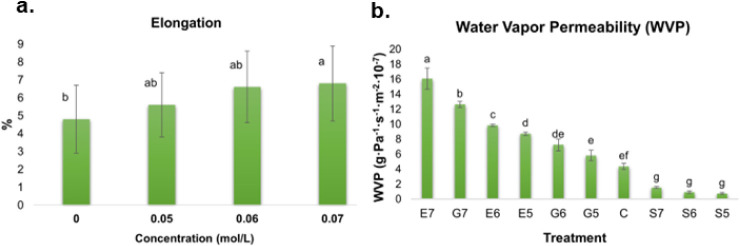
a. Percentage elongation (glycerol at day 1 of storage) and b. water vapor permeability of biopolymer films produced with chayote peels using different plasticizers [glycerol (G), ethylene glycol (E), and sorbitol (S)] at three concentrations [0.05 (5), 0.06 (6), and 0.07 (7) mol/L] with a control (C). Different letters indicate significant differences with α = 0.05. Values are represented as average ± standard deviation (*n* = 3).

The %*E* values of the BFs in this study are comparable to those reported for films developed from other raw materials. For example, Peixoto et al.^68^ reported values ranging from 4% to 8% in films made from potato chip byproducts. De Carli et al.^69^ found %*E* values between 6% and 9% in chitosan-based biodegradable films enriched with polyphenolic propolis residues. Likewise, Chakravartula et al.^70^ observed %*E* values between 3.7% and 24.3% in cassava (*Manihot esculenta* Crantz) starch/chitosan films, while Kamdem et al.^71^ reported values from 2.5% to 4.5% in chitosan-based composite films incorporated with xylan and carvacrol for food applications. This mechanical behavior suggests that the chayote BFs have sufficient extensibility for potential applications in food packaging, where moderate flexibility is essential to prevent cracking or rupture during handling.

The TS at break was 0.22 ± 0.07 MPa for all treatments, with no significant effect of plasticizer type, concentration, storage time, or their interactions (*p* ≥ 0.05). Although the TS values in this research are within the range reported in other studies, these same studies found that plasticizer content had a significant effect on this property. For example, Valderrama-Solano and Rojas de Gante^59^ reported that films made from blue corn flour, plasticized with glycerol and sorbitol at concentrations ranging from 0.3% to 0.45%, showed significant variations in TS, ranging from 0.17 to 0.28 MPa. Similarly, Rompothi et al.^58^ found that edible films based on mung bean starch, plasticized with the same agents at concentrations between 0% and 30%, exhibited TS values ranging from 7.14 to 46.30 ± 3.09 MPa. Moreover, Razavi et al.^67^ observed TS values between 5 and 25 MPa in sage seed gum films containing glycerol and sorbitol at concentrations between 0.3% and 1.5% approximately. Although the TS values of chayote peel-based films were lower, this characteristic makes them suitable for specific applications that do not require high mechanical strength.^72^ These include lightweight food wrapping, temporary protective covers, and flexible packaging where conformability is more important than strength. The mechanical properties of chayote peel films are not yet suitable for load-bearing applications. However, this study aimed to characterize baseline properties and evaluate low-concentration plasticizer effects. These results provide insights into matrix behavior and can guide future improvement strategies, such as cross-linking, biopolymer blending, or plasticizer optimization for packaging applications.

The lack of plasticizer effect in our study may be due to the lower concentrations used, which were significantly lower than those used in the aforementioned studies. Water, present at an average content of 25.52% across all treatments, is a potent plasticizer in BFs and may have masked the effects of added plasticizers.^73^ The absence of a storage time effect on TS may reflect the stability of the polymer network formed in the films.^74^ This stability is advantageous for applications requiring long-term structural integrity, such as food packaging, which must retain its shape and protect its contents during storage and transport.

#### 3.6.5 WVP Analysis

The WVP of BFs is a key factor in assessing product durability, as water transfer through the film, either from the internal or external environment, can cause degradation and a reduce shelf life. For chayote-based BFs, WVP values varied widely, ranging from 7.2 × 10^–8^ to 1.6 × 10^–6^ g·m^–2^·s^–1^·Pa^–1^ (Figure 3b). A significant effect of plasticizer type and concentration on this parameter was observed (*p* < 0.0001), while storage time had no significant effect (*p* < 0.6793). Among the treatments, chayote BFs plasticized with ethylene glycol at 0.07 mol/L showed the highest WVP, followed by those treated with glycerol at the same concentration. Sorbitol-treated films showed the lowest WVP values, with no significant differences among the three concentrations tested. In contrast, BFs with ethylene glycol and glycerol showed increasing WVP with rising plasticizer concentration, a trend previously reported for sweet potato starch films,^60^ kefiran-based films,^75^ and soybean polysaccharide edible films.^11^

This behavior can be attributed to the disruption of the polymer network caused by increasing the plasticizer content, which reduces the intermolecular forces and increases the free volume of the system. As a result, the network becomes less dense, facilitating water vapor transmission.^44,50^ The observed differences in WVP among plasticizers (ethylene glycol > glycerol > sorbitol) are related to their molecular sizes. Ethylene glycol and glycerol, being smaller molecules, penetrate more easily into the polymer matrix, weakening polysaccharide interactions and increasing molecular mobility. In contrast, sorbitol results in a more compact and rigid structure, limiting water diffusion through the film.^76^

Chayote BFs are considered “high barrier films to water vapor”;^77^ however, not all foods require packaging with an extremely low water vapor barrier. Fresh fruits and vegetables, for example, need packaging with intermediate to high WVP in order to maintain proper moisture balance, prevent excessive dehydration, or avoid condensation.^78^ Furthermore, films derived from biological molecules tend to show higher WVP than synthetic materials, which is expected in biodegradable packaging applications based on byproducts.^61^

#### 3.6.6 DSC Analysis

DSC is a key tool for evaluating how temperature variations affect the physical and chemical properties of materials, including possible structural changes or thermal degradation at high temperatures.^79^ In this study, the average Tg of chayote-based BFs was 63.29 ± 1.41 °C, and the average Tm was 154.26 ± 24.73 °C, with an ΔHm of 208.67 ± 25.73 J g^–1^. No significant effects (*p* ≥ 0.05) of plasticizer type, concentration, storage time, or their interactions were found on these thermal parameters. These results differ from other works, such as Oliveira da Silva et al.,^26^ who reported that increasing plasticizer concentration (0.5–0.7 g mL^–1^) decreased Tg, increased polymer chain mobility, and reduced film stability. This discrepancy may be attributed to differences in polymer size, composition, and matrix homogeneity, as well as the lower plasticizer concentration used in our study. Specifically, the plasticizer content in our BFs was less than 1.5%, which may have been insufficient to significantly affect Tg.^80^

Previous studies have reported lower Tg values for various biobased films, such as 22.9–39.7 °C for starch/TiO_2_ films,^64^ 58.2–59.9 °C for poly­(lactic acid)-based films,^81^ and 37.30–51.62 °C for crab chitosan-gelatin films.^82^ A higher Tg is considered beneficial for improving mechanical strength, thermal stability, and durability. Materials with a higher Tg retain stiffness at elevated temperatures, making them more resistant to deformation and wear, an important property for food packaging applications.^83,84^

Only one melting peak was observed in the chayote-based BFs (Figure S1, representative thermogram from replicated measurements), indicating a polymeric matrix without phase separation. This suggests a homogeneous mixture with high compatibility between the components.^85^ In contrast, Kurek et al.^66^ observed a broader Tm range (135.7–169.7 °C), broader peaks, and ΔHm values of 146.4–351.3 J g^–1^ in blackcurrant films. Similarly, Oliveira da Silva et al.^26^ reported Tm values ranging from 135.60 to 159.39 °C and ΔHm values between 109.67 and 176.23 J g^–1^ in bocaiuva flour films. Other studies reported different Tm ranges, such as 172.33–186.67 °C in wheat starch films with TiO_2_ ^64^ and 94.95–102.55 °C in poly­(vinyl alcohol)/gelatin films.^63^

The single, well-defined melting peak in the chayote films, which occurs at a Tm comparable to that of other BFs from natural sources, can be considered a favorable feature. Variations in Tm between different studies are mainly due to differences in chemical composition, molecular size, and degree of homogeneity in the polymeric matrix.^86^

#### 3.6.7 XRD

The XRD pattern of the chayote BFs (Figure 4) exhibited an amorphous character with diffraction peaks at 14.86°, 17.02°, and 22.32°, which are characteristic of pectin and starch.^26,87^ The addition of plasticizers did not significantly modify the peak positions no additional crystalline phases beyond the expected peaks or absence of secondary phase peaks. However, an increase in crystallinity was observed on day 1, as evidenced by a more intense peak compared to the control films. Similar increases in crystallinity have been reported in other starch-based films. For example, Sanyang et al.^57^ determined that increasing plasticizer concentrations (15%–45% sorbitol and glycerol) enhanced crystallinity in sugar palm (*Arenga pinnata*) starch films. Similarly, Zhong and Li^88^ found that crystallinity in kudzu starch films increased with glycerol concentrations up to 40%. Bergo et al.^89^ reported comparable results for cassava starch films with glycerol concentrations ranging from 0% to 45%. In contrast, the present study did not find significant differences in peak intensity between different plasticizer concentrations. This is probably due to the relatively low plasticizer concentrations used and the high water content in the film formulation, with water acting as the main plasticizer.

**Figure 4:**
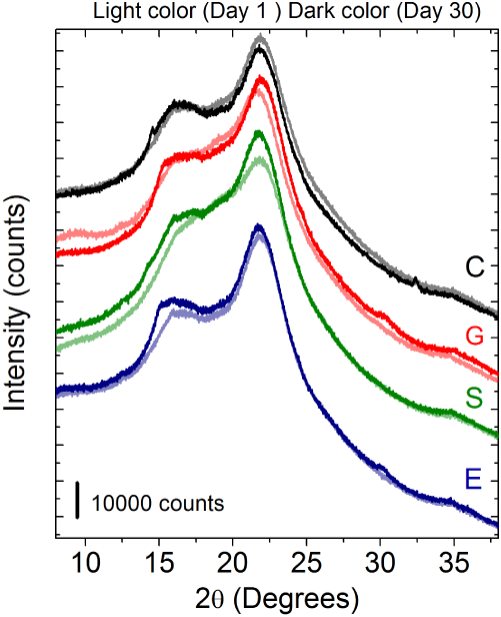
X-ray diffractogram of biopolymer films produced with chayote peel (1 and 30 days) using different plasticizer concentrations [glycerol (G), ethylene glycol (E), sorbitol (S), and control (C)]. Light color (Day 1), dark color (Day 30). Control: black and gray.

When comparing the diffraction patterns between day 1 and day 30 of storage, a decrease in crystallinity was observed in the control BFs. In contrast, the plasticized BFs showed an increase in crystallinity at day 30. This difference may be explained by structural relaxation or water redistribution in the control films, which disrupts the crystalline order. Meanwhile, the presence of plasticizers, such as sorbitol, glycerol, or water likely increases molecular mobility, allowing for better alignment and reorganization of polysaccharide chains, thereby promoting crystallinity. Plasticizers also affect water absorption due to their hygroscopic nature. Over time, this may facilitate the formation of hydrogen bonds and progressive chain reorganization, contributing to a more ordered structure.^90^ Additionally, some plasticizers can act as nucleating agents, promoting the crystallization of specific phases of starch or pectin. In contrast, films without plasticizers tend to maintain lower molecular mobility, limiting reorganization and leading to a decrease in crystallinity.^91^

#### 3.6.8 FTIR-ATR Spectra

The FTIR-ATR spectra of chayote BFs showed absorption peaks in the same regions regardless of plasticizer type or concentration (Figure 5). This suggests that the plasticizers share similar functional groups typical of polyols used in BFs.^57^ The spectra revealed characteristic peaks of starch and pectin. In particular, bands around 1050 cm^–1^ are attributed to C–O stretching of the C–O–C groups on the anhydroglucose ring in starch.^59^ Peaks near 1750 cm^–1^ correspond to stretching of esterified carboxyl (C=O) groups, while peaks between 1600 and 1640 cm^–1^ result from asymmetric stretching of carboxylate (COO^–^) groups found in pectins.^92,93^ Additionally, the peaks around 2900 cm^–1^ are associated with aliphatic C–H stretching in polysaccharides. Broad absorption bands in the 3600–3020 cm^–1^ range correspond to the stretching vibrations of hydroxyl groups from carboxylic acids, absorbed water, and terminal groups in both starch chains and plasticizers.^57^

**Figure 5:**
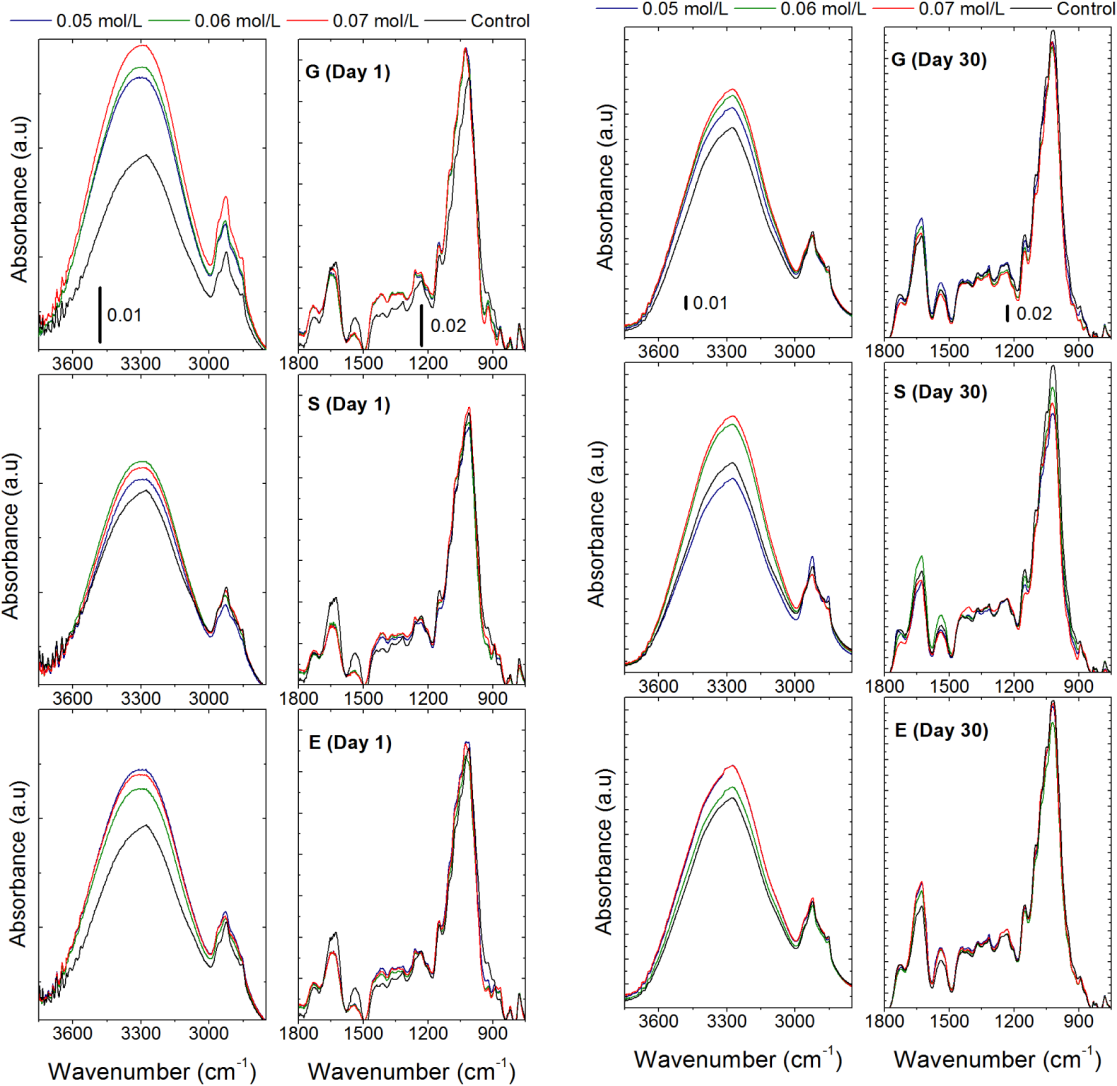
Infrared spectrum (FTIR-ATR) of biopolymer films produced with chayote peel (1 and 30 days) using different plasticizers [glycerol (G), ethylene glycol (E), and sorbitol (S)] at three concentrations (0.05, 0.06, and 0.07 mol/L) and a control (C).

In films containing plasticizers, the −OH (∼3600 cm^–1^) and −CH_2_ (∼2900 cm^–1^) stretching bands were more intense, which can be attributed to the incorporation of polyols rich in −OH groups. Furthermore, variations in the intensity and shape of the −OH stretching region also suggest the formation of intermolecular hydrogen bonds between the −OH groups of the plasticizers or water and the carboxylic or carbonyl groups of the matrix, confirming successful plasticizer incorporation into the film matrix.^94^

After 30 days of storage, the IR spectra of the films still exhibited the characteristic starch and pectin peaks previously described, with no significant changes in intensity. This result is encouraging, as it indicates the structural stability of the material over time. However, in BFs with glycerol, a decrease in the intensity of the ∼3300 cm^–1^ band (associated with O–H stretching) was observed compared to day 1, likely due to a reduction in water-associated −OH groups. In contrast, BFs with sorbitol showed a slight increase in the same region at 0.06 and 0.07 mol/L, suggesting an increase in water content over time. On the other hand, BFs with ethylene glycol exhibited no significant change in this band, indicating a relatively stable water content during storage. This suggests that BFs with this polyol are more stable over time.

These differences in water retention among the plasticizers can be attributed to their hygroscopic properties, molecular size, interactions with the matrix, and degree of plasticization. Glycerol, being small and highly mobile, redistributes water more rapidly; sorbitol, being larger and more rigid, limits water diffusion; and ethylene glycol, with intermediate properties, appears to stabilize water content more effectively. These findings highlight the importance of selecting both the type and concentration of plasticizer to achieve the desired balance of stability and flexibility in chayote-based BFs.^95^

#### 3.6.9 Film Morphology

SEM images of the BFs showed the presence of fibers and particles characteristic of chayote raw material, confirming the integration of this material into the film matrix. The micrographs demonstrated a uniform morphology with no visible phase boundaries, indicating homogeneous distribution without detectable phase segregation. Notable morphological differences were observed between plasticized and nonplasticized films (Figure 6). Nonplasticized BFs exhibited an irregular surface morphology with visible cracks, fractures, and openings (Figure 7 Ca, Cb, and Cc). These structural defects arise from high internal stresses generated during the drying process, where rapid water evaporation creates stress concentration points that exceed the fracture toughness of the material. The brittle nature of unplasticized films is attributable to a dense network of hydrogen bonds between the polysaccharide chains, resulting in limited molecular mobility.^10^ In contrast, BFs with varying concentrations of glycerol, sorbitol, or ethylene glycol showed a more uniform surface morphology free of fractures. These results are consistent with those reported by Sanyang et al.^57^ for sugar palm starch films plasticized with glycerol and sorbitol, where the addition of plasticizer improved surface uniformity. In the present study, the most uniform and smooth microstructures were observed in films prepared with glycerol at 0.05 and 0.07 mol/L, sorbitol at 0.07 mol/L, and ethylene glycol at 0.06 mol/L. This suggests that glycerol, even at lower concentrations, is highly effective in fulfilling its plasticizing role.

**Figure 6:**
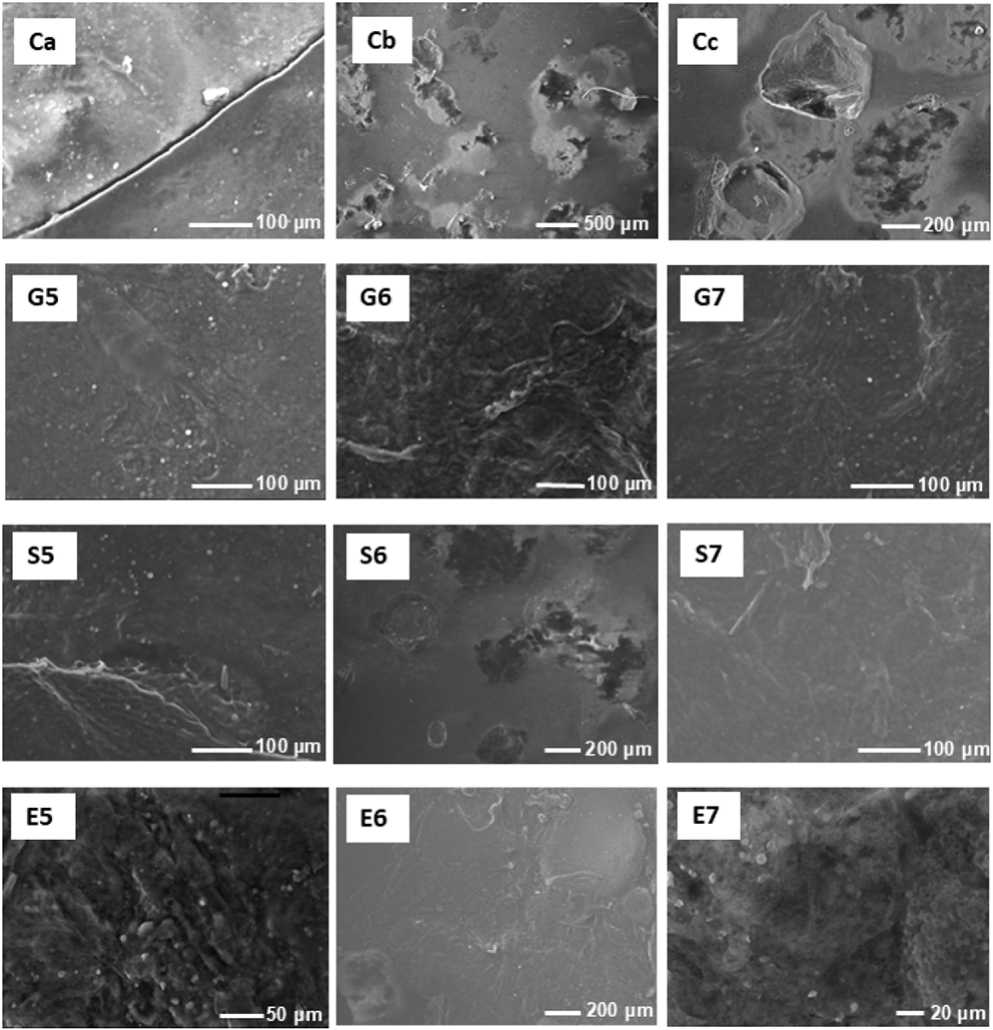
Micrograph of biofilms produced with chayote peels using different plasticizers at day 1. Ca, Cb, and Cc are control treatments, G5, G6, and G7 are glycerol treatments, S5, S6, and S5 are sorbitol treatments, and E5, E6, and E7 are ethylene glycol treatments at three different concentrations (0.05, 0.06, and 0.07 mol/L, respectively).

**Figure 7:**
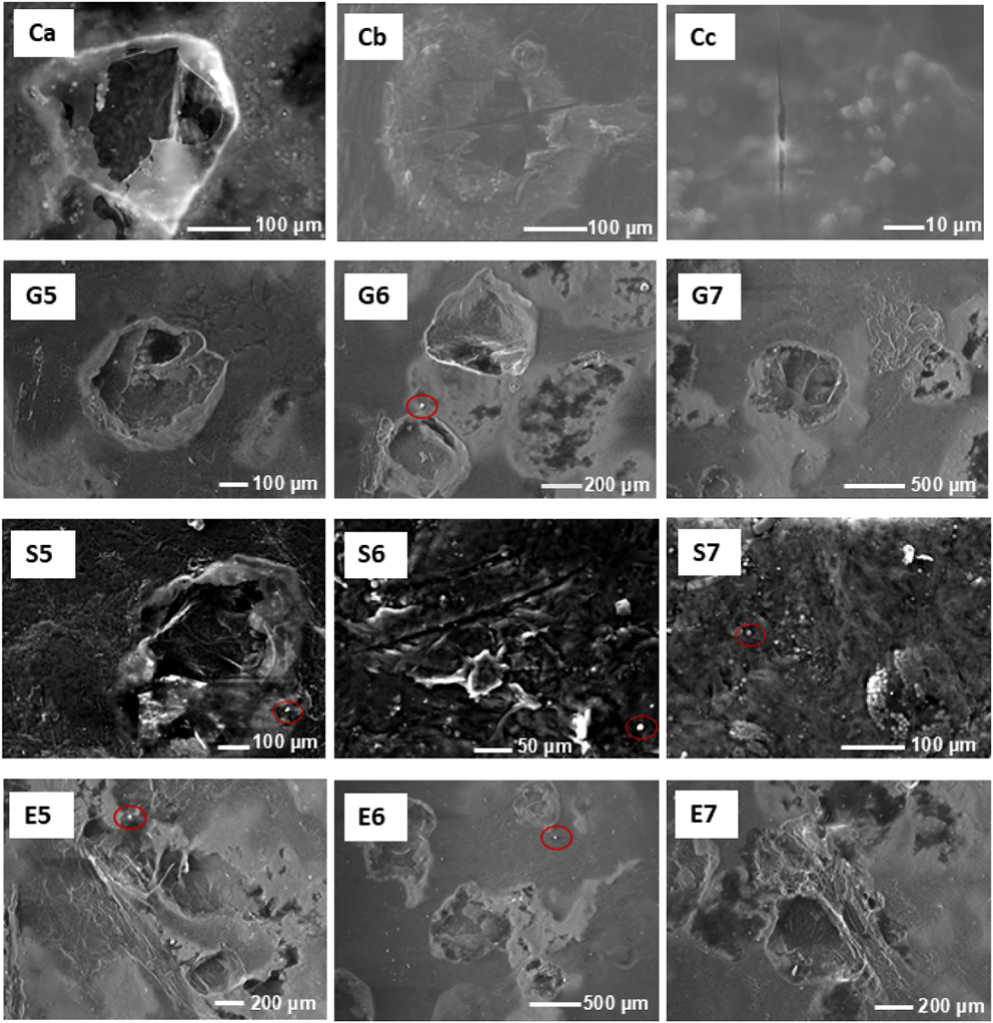
Micrograph of biopolymer films produced with chayote peels using different plasticizers after 30 days of storage at room temperature. Ca, Cb, and Cc are control treatments, G5, G6, and G7 are glycerol treatments, S5, S6, and S7 are sorbitol treatments, and E5, E6, and E7 are ethylene glycol treatments at three different concentrations (0.05, 0.06, and 0.07 mol/L, respectively).

Overall, the results show that plasticizers significantly improved the structural integrity of chayote peel-based films by reducing the presence of cracks and fractures compared to the control films. At the microstructural level, the plasticizers primarily affected the amorphous regions of the polysaccharide matrix, where polymer chain mobility is critical for mechanical performance. The underlying mechanism involves the plasticizers’ hydroxyl groups forming hydrogen bonds with the polysaccharide chains, effectively competing with and weakening the existing polymer–polymer hydrogen bonding network. This molecular disruption increases the free volume between chains and reduces intermolecular forces, thereby enhancing chain mobility and flexibility.^96^

In the case of polyol plasticizers like glycerol, sorbitol, and ethylene glycol, their multiple hydroxyl groups enable them to establish extensive hydrogen bonding with the polysaccharide backbone, effectively replacing rigid polymer–polymer interactions with more flexible polymer-plasticizer interactions.^97^ This molecular rearrangement transforms the microstructure from a tightly packed, brittle network into a more loosely organized, flexible matrix. This effect can be attributed to the plasticizers’ ability to increase matrix flexibility by weakening intermolecular interactions between polymer chains. As a result, the stiffness of the material is reduced while its %*E* are increased, reducing the risk of fractures in the BFs.^98^ However, after 30 days of storage, most of the plasticized BFs exhibited an increased number of ruptures (Figure 7). This phenomenon can be attributed to physical aging processes, characterized by structural relaxations that naturally occur when amorphous polymeric materials are stored below their glass transition temperature.^99^ Interestingly, the treatment with sorbitol at 0.07 mol/L showed the fewest ruptures after 30 days of storage. This superior performance can be explained by the molecular structure of sorbitol, which contains six hydroxyl groups compared to the three in glycerol, providing greater hydrogen bonding capacity with both the polymer matrix and water molecules. At this maximum concentration, sorbitol appears to have effectively preserved the films’ moisture content, achieving an optimal balance between moisture retention and flexibility, thus reducing their fragility during prolonged storage.

Additionally, crystalline formations were observed in some areas of the stored films [Figure 7 (red circles mark the crystalline formations) and Figure S1]. EDX spectroscopy analysis revealed that the crystals contained mainly carbon, oxygen, chlorine and potassium (as indicated in Figure S2 by their characteristic Kα emission lines: C Kα, O Kα, Cl Kα, and K Kα, respectively). According to Xie et al.,^100^ crystal formation in films made from agricultural byproducts is generally attributed to impurities or residues of specific compounds that crystallize under certain storage conditions or result from chemical reactions among film components or residual substances introduced during processing. While crystallization can improve certain film properties, it can also reduce barrier effectiveness over time.^101^ This duality underscores the importance of optimizing film composition and storage conditions to achieve desirable performance in food preservation applications.^72^

## 4 Conclusions

This study shows the potential that chayote peels have to be used as a sustainable base for developing BFs. The resulting films were homogeneous, continuous, and stable, with colors ranging from cream to light brown. The addition of the plasticizers glycerol, sorbitol, and ethylene glycol differentially affected the physicochemical and mechanical properties of the films. They improved flexibility and malleability, reduced brittleness, and facilitated demolding. They also improved the brightness and color intensity of the films, probably by increasing light reflection. Regarding physicochemical properties, thickness, water solubility, and resistance to acid and basic solutions did not show significant changes depending on plasticizer type or concentration. Mechanically, %*E* increased with higher plasticizer concentrations, while TS remained stable. WVP varied with plasticizer: ethylene glycol and glycerol increased permeability, while sorbitol reduced it. Thermal analysis by DSC showed no significant differences in thermal stability, indicating a homogeneous matrix. XRD analysis revealed increased crystallinity in plasticized films over time, suggesting structural rearrangement promoted by the plasticizers. SEM observations confirmed improved film morphology, with smoother and more uniform surfaces in plasticized samples. Overall, the results highlight the importance of selecting the appropriate plasticizer type and concentration to optimize the functional properties of chayote peel-based films, particularly in terms of flexibility, moisture retention, and storage stability. While this study demonstrates laboratory-scale feasibility, industrial scaling requires optimizing drying parameters, thickness uniformity, and barrier properties. Future research should explore alternative processing techniques and assess commercial viability, as well as biodegradability or composting studies.

## Supplementary Material



## Notes

Figure S1: Differential scanning calorimetry (DSC) representative thermogram of biopolymer films produced with chayote peel showing the glycerol 0.05 mol/L sample; Figure S2: Energy-dispersive X-ray analysis of crystals found in chayote biopolymer films at 30 days of storage (PDF)
