# Supplementary material for: Effect of Polyol Plasticizers on the Physicochemical, Mechanical, and Microstructural Properties of Films from Chayote (Sechium edule) Peels
Source: ACS Omega. 2026 Feb 4;11(6):9076–89. doi: 10.1021/acsomega.5c07160 (PMC12917696; doi:10.1021/acsomega.5c07160)
Supplement: Supplementary file 1 [file ao5c07160_si_001.pdf]

Effect of polyol plasticizers on the physicochemical, mechanical, and microstructural properties of films from chayote (*Sechium edule*) peels

Laura Arroyo-Esquivel[*,1,2], Víctor M. Jiménez[3], Fabián Vásquez-Sancho[4], Esteban Avendaño-Soto[4], Patricia Esquivel[1,2]

[1]Escuela de Tecnología de Alimentos, Universidad de Costa Rica, 11501-2060 San Pedro, Costa Rica.

[2]Centro Nacional de Ciencia y Tecnología de Alimentos (CITA), Universidad de Costa Rica, 11501-2060 San Pedro, Costa Rica.

[3]Centro para Investigaciones en Granos y Semillas (CIGRAS) and Instituto de Investigaciones Agrícolas (IIA), Universidad de Costa Rica, 11501-2060 San Pedro, Costa Rica.

[4]Escuela de Física and Centro de Investigación en Ciencia e Ingeniería de Materiales, Universidad de Costa Rica, 11501-2060 San Pedro, Costa Rica.

*Correspondence to: laura.arroyoesquivel@ucr.ac.cr

Laura Arroyo-Esquivel: https://orcid.org/0009-0005-8888-0229,

Víctor M. Jiménez: https://orcid.org/0000-0003-3771-6072,

Fabián Vásquez: https://orcid.org/0000-0001-8814-2676,

Esteban Avendaño: https://orcid.org/0000-0001-5500-3821

Patricia Esquivel: https://orcid.org/0000-0002-2555-9535

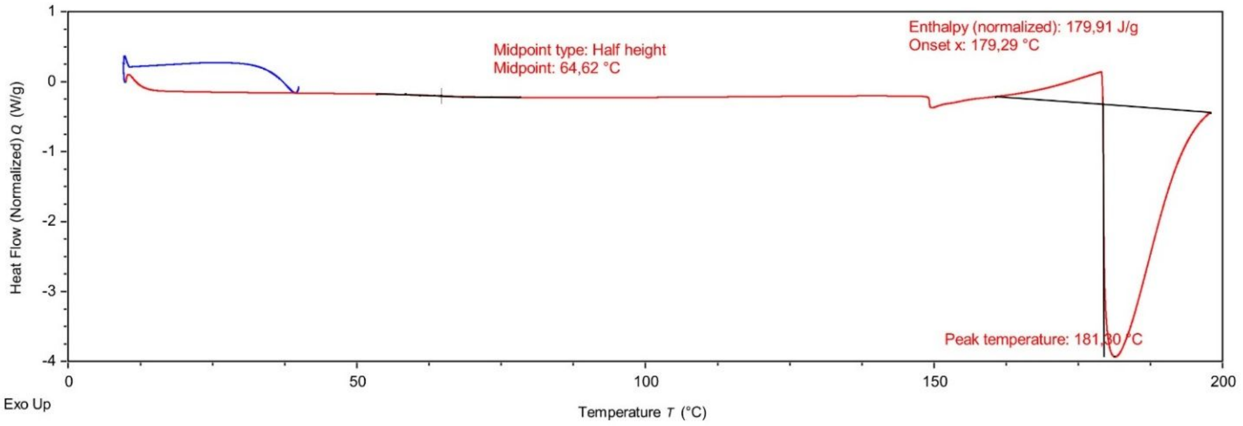

Figure S1. Differential scanning calorimetry (DSC) representative thermogram of biopolymer films produced with chayote peel showing the glycerol 0.05 mol/L sample.

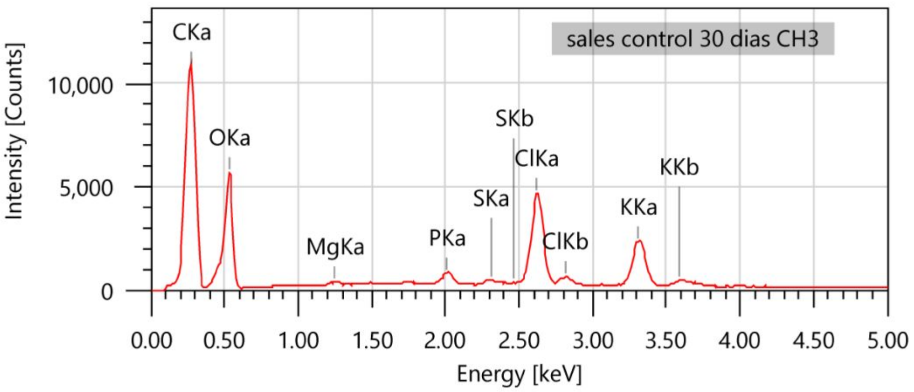

Figure S2. Energy dispersive X-ray analysis of crystals found in chayote biopolymer films at 30 days of storage.
